# Canada

**Published:** 1896-05

**Authors:** 


*Canada.* The House of Parliament at Ottawa, on March 27th, vigorously denounced the action of Great Britain in placing such restrictions on Canadian cattle as now exist, on the ground of receiving from that country cattle affected with contagious pleuro-pneumonia. Mr. Mulock in closing his remarks said forcibly: "Your contention is that you are keeping Canadian cattle out because of the existence of contagious pleuro-pneumonia in Canada. We respectfully submit to you as our united opinion that no pleuro-pneumonia exists in Canada, and consequently that your basis for legislation is not a good basis." Sir Charles Tupper reviewed the subject from the first scheduling in 1892, and ventilated thoroughly the unstable grounds and unfair position assumed by the British authorities on this matter. He well asked why was not the offer of Canada accepted, when she invited the mother-country to send her highest veterinary authorities to investigate on Canadian soil at the latter's expense, to satisfy themselves that such a disease was unknown to them as a cattle-growing country, and we might also add, what an anomalous position for a people to take, when for the past ten years their own country had been the worst pest-centre for the existence and perpetuation of this disease known. Why will not the government of Great Britain be honest with itself and fair with sister-countries, and say, We do not want your meat-products, not because they are not good, not because they are not cheaper than our own, but for no other reason than that their introduction will interfere with the business of our home raisers of cattle.

---

A WHITE colt, with red ears, by Palentine, has been dropped at Oak Hill Farm, near Nashville, and hundreds have visited the equine freak.

RECENT researches into the birth of 16,000 foals show that 97 colts were born to every 100 fillies; that up to the end of the third month of gestation the fœtus is sexless, and the sex is determined by the bodily condition of the mother in the fourth and fifth months. If the mare and fœtus be well nourished, the sex is likely to be a female.

